# Para além dos centros: a ciência norte-americana no Brasil do século XIX

**DOI:** 10.1590/S0104-59702026000100021

**Published:** 2026-06-29

**Authors:** Olivia da Rocha Robba

**Affiliations:** i Professora, Universidade Federal da Fronteira do Sul. Laranjeiras do Sul – PR – Brasil. prof.oliviarobba@gmail.com


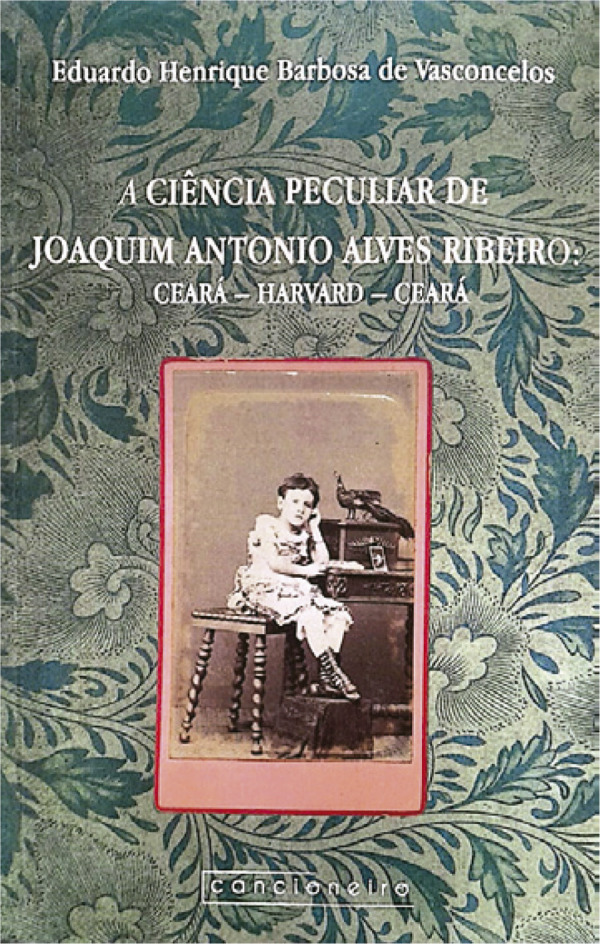


O livro de Eduardo Henrique Barbosa de Vasconcelos, professor da Universidade Estadual de Goiás, é resultado da sua pesquisa de doutorado em história social, defendida em 2023, no Programa de Pós-graduação em História da Universidade Federal do Rio Grande do Sul. O livro trata da formação e das atividades científicas do médico e naturalista cearense Joaquim Antônio Alves Ribeiro (1830-1875), primeiro brasileiro nato a obter o título de médico por Harvard, em 1853.

O estudo sobre a trajetória de Joaquim Antônio Alves Ribeiro aprofunda uma discussão iniciada em sua dissertação de mestrado, na qual já expressava sua crítica contra a tradição histórica e “explicações fatalistas que priorizam a seca, o cangaço e o messianismo como únicos aspectos válidos para as explicações construídas para o entendimento da história social, econômica, política e cultural do Ceará” (Vasconcelos, 2007, p.6), que centralizam e obliteram a possibilidade de novas perspectivas acerca de sua história.

Sua pesquisa dialoga com as reflexões propostas por Durval Muniz de Albuquerque Jr. (2009, 2012), que apresenta uma importante inflexão acerca da construção do Nordeste no imaginário brasileiro, chamando atenção para o preconceito de origem e de lugar que afeta o povo nordestino. O autor se insere nesse debate ao tirar do anonimato a trajetória do médico e naturalista cearense e trazer novos elementos para uma reflexão crítica sobre o papel reservado, em nossa historiografia, para a província do Ceará ao longo do século XIX.

Dois pontos foram basilares para o desenvolvimento de seu trabalho: o primeiro deles é o Gabinete de História Natural do Ceará, criado por iniciativa de Joaquim Antônio Alves Ribeiro na segunda metade do Oitocentos e que, mais tarde, foi doado à província, passando a ser chamado de Museu de História Natural do Ceará; o segundo é o detalhamento das práticas científicas realizadas pelo médico cearense ao longo da sua vida.

Inicialmente o autor faz um balanço historiográfico sobre a história do Ceará e sua inserção na história das ciências no Brasil, chamando atenção para os principais aspectos, estereótipos e silenciamentos que reforçam a ideia equivocada de que o desenvolvimento de práticas científicas eram incompatíveis com as condições materiais oferecidas pelas províncias mais afastadas dos grandes centros de poder no Império do Brasil. Dessa forma, tece uma crítica importante no que tange aos silenciamentos provocados por uma historiografia que pensa o Brasil a partir do Sudeste, centro irradiador do poder político e econômico.

Ao longo do livro é apresentada a trajetória científica do doutor Alves Ribeiro, com destaque para sua formação em medicina em Harvard e a construção da sua coleção particular, que constituía o Gabinete de História Natural, salientando os diferentes aspectos do processo de institucionalização dos museus no Brasil. Posteriormente, essa coleção passou a compor o acervo do Museu de História Natural do Ceará, instituição científica cujos primeiros exemplares remontam a um período que antecedeu à criação das primeiras universidades no Brasil. Tal como destaca Maria Amélia M. Dantes (2001), trata-se de um espaço privilegiado que desempenhou papel relevante na implementação de atividades científicas no país.

O autor também destaca o perfil do médico Alves Ribeiro, homem de ciência do seu tempo, e problematiza as principais atividades desempenhadas ao longo da sua trajetória: a correspondência com a Boston Society of Natural History; a análise da qualidade da água do açude Pajeú; a publicação do *Manual da parteira* e do jornal *A Lanceta*, além do debate sobre o uso do éter como anestésico, por meio do instrumento científico chamado pelo doutor Alves Ribeiro de “insensibilizador”.

Para tanto, Eduardo Vasconcelos (2024) utilizou teses, dissertações, artigos e ampla bibliografia sobre o tema, além de periódicos, manuais técnicos, relatórios provinciais e documentos de época, que dão um grande suporte empírico a sua pesquisa e contribuem imensamente para as discussões sobre as práticas científicas desenvolvidas nas margens do Império ao longo do Oitocentos, em especial, no Ceará.

Juntamente com a publicação do livro, o autor criou um *site* (https://alvesribeirocientista.com.br/) disponibilizando informações tanto sobre os resultados de sua pesquisa como a respeito do médico cearense.

Por fim, sobressai a atualidade do estudo que reflete criticamente sobre o lugar das atividades científicas realizadas no espaço geográfico atualmente identificado como Nordeste, na historiografia da história das ciências no Brasil, chamando a atenção sobre a produção de conhecimento e práticas científicas fora dos grandes centros ao longo do século XIX. Além disso, a obra nos leva a refletir criticamente sobre as limitações dos modelos eurocêntricos que, até alguns anos atrás, foram hegemônicos na academia brasileira, atribuindo pouca importância aos saberes produzidos em outras regiões, como a África, a Ásia, a América Latina e os Estados Unidos, onde Alves Ribeiro estudou e aprendeu a ciência que passou a desenvolver no Ceará. Tudo isso ocorreu e ainda ocorre seja pelo desinteresse dos historiadores, seja pela força do mito do vazio científico ou da escassez, que ainda persiste na historiografia.
